# Tobacco as a Disinfectant

**Published:** 1906-07

**Authors:** 


### Tobacco as a Disinfectant.

Although there is a general impression that tobacco smoke is a germicide, this property has not been assigned hitherto to any one particular constituent of the smoke. The author of a note on the subject in *The Lancet* (London, April 2) gives reason for believing that among other germicidal constituents the smoke contains formaldehyde. He says:

"The composition of tobacco smoke is, of course, complex, but everybody knows that tarry oils are a principal constituent, and certainly many oils are powerfully antiseptic. Nicotin, again, is a strong disinfectant, but the quantity of this poison in tobacco smoke is minute, if, indeed, it is present at all. At least, in a number of chemical analyses of tobacco smoke made at different times it was difficult to declare with absolute certainty that nicotin was an important constituent. The oily matter which accumulates in a tobacco pipe is decidedly poisonous, but it does not contain any appreciable quantity of nicotin, the chief constituent being the very poisonous oil pyridin. Tobacco smoke contains a decided quantity of the very poisonous gas carbon monoxid which has been used for preserving purposes and which, therefore, must possess germicidal properties. Some simple experiments which we have recently made would seem to confirm the observation that one of the principal constituents accounting for the germicidal properties of tobacco smoke is the powerful antiseptic formaldehyde. The amount present is more than just appreciable, for if water through which a few puffs of tobacco smoke have been passed is tested for formaldehyde the result is strikingly positive. The quantity of formaldehyde in tobacco smoke would appear to depend on the quality and kind of tobacco smoked. Thus the cigar appears to yield more formaldehyde than the pipe, and the pipe more than the cigarette. Possibly the peculiarly irritating property of the smoke issuing from the glowing end of a cigarette, cigar or from the bowl of a pipe is due to formaldehyde. It has more than once been stated that tobacco smokers enjoy an immunity from certain diseases; and the frequent presence of a powerful antiseptic in the mouth, nasal passages, and sometimes the lungs (as in the case of those who foolishly inhale tobacco smoke) would to some extent justify the statement. When it is considered that in the nose a vast number of microbes are hourly deposited it is conceivable that these may be effectively destroyed by the frequent passage of tobacco smoke through that organ. In the same way the organisms exposed to tobacco smoke in the mouth would succumb. Formaldehyde is one of the most powerful disinfectants we possess, 1 part in 10,000 parts of water serving to destroy all microbes, while such a dilute solution has practically no poisonous action on the human organism. All the same, it is most undesirable that this fact should stimulate the practice of smoking tobacco to absurd excess, for tobacco poisoning is a greater reality than many persons think, and to employ tobacco in abusive quantities for the sake of destroying microbes might amount possibly to killing the seeds of one disease only to contract another."—*Literary Digest.*

